# Raman study of colloidal Cu_2_ZnSnS_4_ nanocrystals obtained by “green” synthesis modified by seed nanocrystals or extra cations in the solution

**DOI:** 10.1016/j.heliyon.2023.e16037

**Published:** 2023-05-06

**Authors:** O.A. Kapush, V.M. Dzhagan, N.V. Mazur, Ye.O. Havryliuk, A. Karnaukhov, R.A. Redko, S.I. Budzulyak, S. Boruk, I.S. Babichuk, M.I. Danylenko, V.O. Yukhymchuk

**Affiliations:** a V. Lashkaryov Institute of Semiconductors Physics, National Academy of Sciences of Ukraine, 45 Nauky Av., 03028, Kyiv, Ukraine; b Physics Department, Taras Shevchenko National University of Kyiv, 60 Volodymyrs'ka Str., 01601, Kyiv, Ukraine; c Semiconductor Physics, Chemnitz University of Technology, D-09107, Chemnitz, Germany; d Center for Materials, Architectures, and Integration of Nanomembranes (MAIN), Chemnitz University of Technology, D-09107, Chemnitz, Germany; e State University of Telecommunications, 7 Solomenska Str., 03680, Kyiv, Ukraine; f Yurii Fedkovich Chernivtsi National University, 25, Lesia Ukrainka Str., 58000, Chernivtsi, Ukraine; g Faculty of Intelligent Manufacturing, Wuyi University, Jiangmen, 529020, PR China; h Frantsevich Institute for Problems of Materials Science, National Academy of Sciences of Ukraine, Kyiv, Ukraine

**Keywords:** Semiconductor nanocrystals, Colloidal synthesis, Cu_2_ZnSnS_4_, Raman spectra, Phonons

## Abstract

The method of affordable colloidal synthesis of nanocrystalline Cu_2_ZnSnS_4_ (CZTS) is developed, which is suitable for obtaining bare CZTS nanocrystals (NCs), cation substituted CZTS NCs, and CZTS-based hetero-NCs. For the hetero-NCs, the synthesized in advance NCs of another material are introduced into the reaction solution so that the formation of CZTS takes place preferably on these “seed” NCs. Raman spectroscopy is used as the primary method of structural characterization of the NCs in this work because it is very sensitive to the CZTS structure and allows to probe NCs both in solutions and films. Raman data are corroborated by optical absorption measurements and transmission electron microscopy on selected samples. The CdTe and Ag NCs are found to be good seed NCs, resulting in a comparable or even better quality of the CZTS compound compared to bare CZTS NCs. For Au NCs, on the contrary, no hetero-NCs could be obtained under the given condition. Partial substitution of Zn for Ba during the synthesis of bare CZTS NCs results in a superior structural quality of NCs, while the introduction of Ag for partial substitution of Cu deteriorates the structural quality of the NCs.

## 1 Introduction

Photovoltaics is one of the most promising ways to affordable alternative energy supply, and the intensity of research work in this area is steadily increasing. The materials for the efficient absorber layer of a solar cell must possess a suitable bandgap and a high coefficient of light absorption in the spectral range of solar radiation, high mobility of the photogenerated charge carriers, non-expensive and environmentally friendly constituents and fabrication technologies, as well as compatibility with the thin-film deposition techniques. The family of quaternary metal chalcogenides like Cu_2_ZnSnS_4_ (CZTS) has been intensively studied in the past decade as a promising candidate for various solar cell architectures [1,2]. The CZTS has a suitable bandgap (E_g_ ≈ 1.46 eV, and can be tuned to longer wavelength by alloying with Selenium), a rather high absorption coefficient (α > 10^4^ см^−1^), consists of non-expensive and non-toxic elements, and exhibits a favorable energy band alignment with popular semiconductors and oxides, promising not only for photovoltaic but also for other applications [[1], [2], [3], [4], [5]].

There has been continuous work on modification of the CZTS-like materials in order to increase their photovoltaic [[6], [7], [8]], photocatalytic [5], or thermoelectric efficiency [3,4] through the elimination of high concentration of structural defects, especially in the cationic sublattice. In particular, a partial cation substitution (Cu for Ag, Zn for Ba, or Sn for Ge) has been investigated intensively in this respect [[5], [6], [7], [8], [9], [10], [11]], although this approach did not lead yet to a noticeable advance in the efficiency in the above applications of the CZTS-like materials.

Nanocrystals (NCs) synthesized using colloidal chemistry are very promising for all the above applications of the CZTS and similar NCs [2]. These methods enable reasonable control of the material properties and allow the deposition of thin films on deliberate surfaces/substrates, as well as the formation of the NC composites with polymers or other nanostructures [2,[12], [13], [14]]. However, the properties of the NCs obtained employing colloidal synthesis are dependent on many parameters, such as temperature, the nature of the dispersion medium (solvent) and stabilizer (ligand), the concentration of the reagents, pH of the solution, as well as post-synthesis treatments deposition of thin films. Therefore, a detailed study of all these factors and their possible interrelation is required for obtaining the technology of NCs with controllable and reproducible characteristics [[15], [16], [17], [18]].

In contrast to intensively investigated cationic and anionic substitution in CZTS NCs (in hundreds of publications) [2,5,10,[19], [20], [21], [22]], very few studies have been reported so far on CZTS-based hetero-NCs [11,[23], [24], [25], [26], [27]]. Although this area of NC research has been extremely active for their II-VI [13,[28], [29], [30]] and I-III-VI [31] “nano-relatives” in the past decade. Among numerous employed characterization techniques, Raman spectroscopy became an established characterization tool for CZTS-like and many related multinary semiconductors, including colloidal NСs [[31], [32], [33], [34], [35], [36], [37]], because it can provide both the chemical composition and strain [31], can detect secondary (impurity) phases [35,[38], [39], [40]] and even point defects [33,41,42]. Moreover, it does not require a large amount of material, unlike X-ray diffraction (XRD), or special sample preparation for the measurement, unlike transmission electron microscopy (TEM). Moreover, it can probe NCs even in as-synthesized solutions. Therefore, Raman spectroscopy was chosen in this work as the primary characterization tool of the CZTS-based NCs synthesized using an affordable and non-toxic route in aqueous media.

## 2 Material and methods

### 2.1 NC synthesis

*Preparing the Sn precursor solution.* A stock aqueous 0.5 M solution of SnCl_2_ in 4.0 M NaOH was prepared by slowly pouring an aqueous 1.0 M suspension of SnCl_2_ × 2H_2_O into an aqueous 8.0 M solution of NaOH (volume ratio of the suspension and NaOH solution was 1:1).

*Synthesis of CZTS NCs.* The colloidal CZTS NCs were synthesized as a result of a reaction between the mixture of thio-complexes of Cu^2+^, Zn^2+^, Sn^2+^, and Na_2_S in water. In a basic synthesis procedure (if not specified differently), 0.3 ml of aqueous 1 М solution of Cu(NO_3_)_2_, 0.3 ml of aqueous 0.5 М solution of SnCl_2_ (with 4.0 М NaOH), and 0.15 ml of aqueous 1 М solution of Zn(CH_3_COO)_2_ were added to 8.28 ml of deionized water at continuous stirring, with subsequent adding of 0.22 ml of thioglycolic acid (TGA) and 0.32 ml of aqueous 1 M solution of NaOH. Finally, to the obtained solution, we added 0.3 ml of aqueous 1 M solution of Na_2_S, and the resulting final solution was subject to heating at 95–98°С for 10 min.

One of the key differences (and novelty) of our synthesis route from that in the original approach to the synthesis proposed in Ref. [43] is using pure TGA instead of its solution. This modification was inspired by our experience in the synthesis of other colloidal NCs using TGA as a ligand, in particular CdTe [44], for which NCs of better structural quality were obtained with using pure TGA compared to using TGA solution. Another important difference of our approach from the protocol used in Ref. [43] is that instead of freshly prepared at 70–80 °C SnCl_2_ solution in NaOH we used such a solution prepared at a much lower temperature, 40–45 °C, and waited until the tin hydroxide dissolves completely (at least 24 h).

*Synthesis of CZTS-based cation-substituted and hetero-NCs*. For the formation of cation substituted and hetero-NCs, the corresponding cations and seed NCs of other compounds were added into the reaction medium in the ratio indicated in the corresponding figures discussed in the main text of the manuscript. In particular, for the synthesis of CZTS:Ag NCs, 1 М solution of AgNO_3_ was added from 0 to 0.3 ml. For obtaining CZTS:Ba NCs, 1 М solution of BaCl_2_ was added from 0 to 0.3 ml. The synthesis of CdTe NCs used in this work was described by us earlier [15,18,44]. The Ag and Au NCs were synthesized by the well-known AgNO_3_ or AuHCl_4_ reduction with sodium citrate [45,46].

### 2.2 Characterisation

Raman spectra were excited with a 532 nm single-longitudinal-mode solid-state laser, with a power density on the samples of less than 10^3^ W/cm^2^, which was low enough to preclude any thermal or photo-induced modification of the sample. Dispersion of the spectra was performed using a single-stage spectrometer (MDR-23, LOMO) with a spectral resolution of 3 cm^−1^ (as measured by the peak width of single crystal Si substrate and Rayleigh peak). Detection of the spectra was performed with a TE-cooled (- 60 °C) CCD detector (Andor iDus 420). At least 4 spots were probed in each sample, to ensure that the sample is homogeneous and the obtained spectra are representative. Optical absorption spectra were recorded from NC solutions using the StellarNet Silver Nova 25 BWI6 Spectrometer. TEM images were obtained with a JEM-2100F microscope; an acetone suspension of the NCs was subject to ultrasonic treatment and applied on a copper grid covered with an ultrathin carbon film.

## 3 Results and discussion

### 3.1 CZTS synthesized in presence of CdTe and Ag seed NCs

The general approach to the facile and “green” route of the CZTS NC synthesis in aqueous solutions was developed in recent work [43]. In the present work, it is used with several modifications, as described in the *Experimental section*, and extended to the formation of the CZTS on “seed” NCs of other materials, in particular CdTe, Ag, and Au, which heterojunctions that can be of interest from an application point of view. In addition, the effect of a partial cation substitution of Cu for Ag (CZTS:Ag) or Zn for Ba (CZTS:Ba) was investigated.

**Fig. 1:**
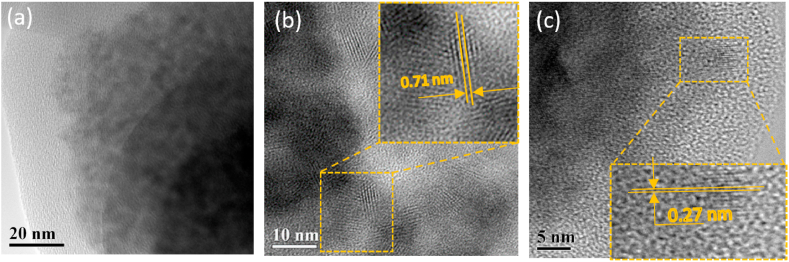
TEM images of CZTS (a), CdTe (b), and CdTe/CZTS NCs (c).

According to the detailed TEM and XRD studies performed in Ref. [43], this type of synthesis results in a NCs size spreading from about 2 to 7 nm, with a mean size of 4 nm. The TEM study of the bare CZTS NCs synthesized in this work is in agreement with that previous study and also reveals particles with sizes below 10 nm (Fig. 1a). In the similar size range are also the CdTe NCs used here as one of the types of “seed” NCs for subsequent CZTS synthesis (Fig. 1b). Contrary to CZTS NCs, the CdTe NCs exhibit the clear structure of the crystal lattice, with the interplanar distance 0.71 nm correlating well with those reported before for cubic CdTe (0.648 nm) [47]. This is in agreement with a generally known tendency to high crystallinity of the II-VI NCs (even when synthesized at low temperature, <100 °C) [13,15,18,22,28], in contrast to CZTS-like compounds which suffer from various types of structural disorder and defects even when synthesized at high temperatures (>300 °C) [33,39,41,42,48]. In the TEM images of the heterogeneous CdTe/CZTS NCs (Fig. 1c), the crystalline structure of the CdTe NCs is not seen, indicating the formation of the CZTS phase on them. This assumption is additionally confirmed by observing fringes of the crystalline lattice with the interplanar distance of 0.27 nm, which is close to the distances between the (112) planes of kesterite CZTS (0.31 nm) reported before [49]. The obtained results indicate that the crystalline structure of CdTe nanoparticles can effectively create conditions for appearing of the crystalline phases of CZTS.

**Fig. 2:**
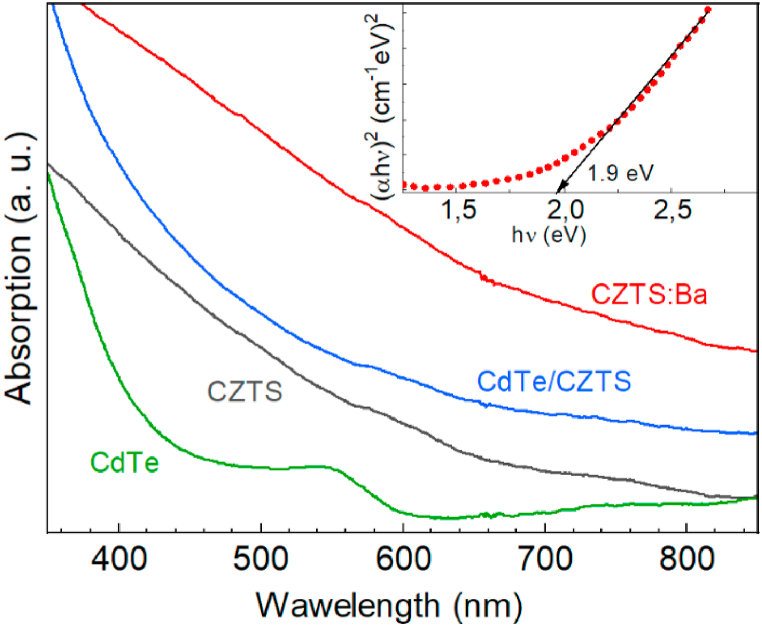
Representative optical absorption spectra of different types of NCs investigated in this work. The inset shows a presentation of the CZTS:Ba spectrum in the form of a Tauc plot.

The representative optical absorption spectra of different types of NCs studied in this work are shown in Fig. 2. Contrary to the spectrum of CdTe NCs, which typically for II-VI NCs exhibits a sharp absorption onset and absorption maximum due to the lowest-energy interband transition [13,29], the spectra of other types of NCs, which contain CZTS, are featureless. This lineshape is common for different ways of CZTS synthesis, as well as for related metal chalcogenides with inherent types of structural disorder, such as cation vacancies [50] and cationic disorder [35]. Therefore, the optical spectra are not informative regarding the structure of the CZTS and are based on its heterostructures.

**Fig. 3:**
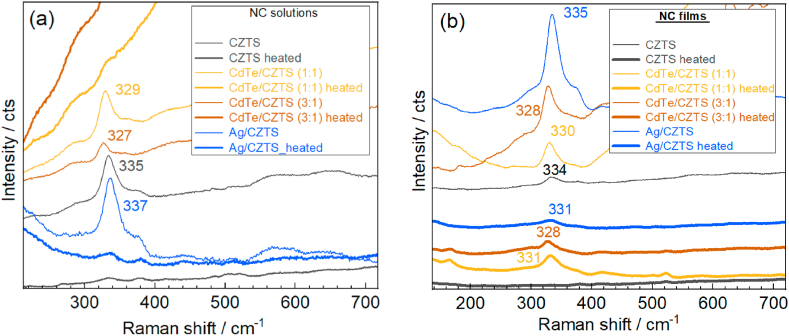
Raman spectra (λ_exc_ = 532 nm) of CZTS and hetero-NCs synthesized at 10–14 °C, measured in solution (a) and dried film (b). Thin curves correspond to as-synthesized samples, and thick curves of the same color – corresponding sample subject to post-synthesis heating.

Previously, Raman spectroscopy has been shown to be one of the most informative characterisation methods regarding the structural and elemental composition of CZTS-like compounds [[32], [33], [34], [35]]. The main spectral fingerprint of the CZTS structure is the (A_1_-symmetry) phonon peak in the range of 320–340 cm^−1^. The occurrence of this vibration at larger frequencies, 336-339 cm^−1^, is characteristic of the ordered kesterite structure, while the lower values, 326-332 cm^−1^, are commonly attributed to so-called disordered kesterite (*i.e.* with antisite occupation of Cu and Zn ions) [48,[51], [52], [53]]. In the Raman spectra of the series of hetero-NCs obtained in this work (Fig. 3), measured at λ_exc_ = 532 nm that is resonant for the excitation of the phonons in CZTS [34], this peak is present in all the spectra of as-synthesized NCs (not subject to post-synthesis thermal treatment). In the bare CZTS NCs, the Raman peak position exhibits an intermediate position between the abovementioned typical for ordered and disordered cation structures, which is a situation often observed for CZTS NCs synthesized at mild conditions [21,35,38,43,53,54].

In the case of CZTS synthesis in the presence of CdTe NCs, a larger concentration of seeds leads to the formation of a smaller volume of CZTS, as concluded from the lower Raman peak intensity. Moreover, the lower frequency of the mode, 327 vs 329 cm^−1^, may indicate more cation disorder in the kesterite structure formed on a larger concentration of CdTe seeds and or strain induced by lattice mismatch with CdTe. It should be noted that in the context of the cation disorder in kesterite the larger magnitude of the “disorder” is not necessarily accompanied by a larger FWHM of the peak [32,42]. The main indication of the “disorder” (*i.e.* antisite occupation of Cu and Zn ions) is the peak frequency, as we can also see in the spectra of this work. Note that Raman spectra are a good probe also for secondary (impurity) phases that can form during the synthesis of CZTS [38], and at visible excitation, it is especially sensitive to Cu_x_S [34,35]. However, no indications of the Cu_x_S – expected around 470 cm^−1^ or other secondary phases [34,35] can be seen in our spectra.

As to the CZTS NCs synthesized in the presence of Ag NCs, the characteristic kesterite peak (intensity, position, and FWHM) is very close to that of bare CZTS NCs.

All the effects observed above on the NCs in the solutions are also qualitatively preserved on the dried NC films deposited by the drop-casting onto the glass substrate (Fig. 3b).

A frequently used step of colloidal synthesis of NCs is a post-synthesis thermal treatment (heating) of the NCs in the solution. This step was also employed in work on CZTS NC synthesis [43], which we used as a starting point for our synthesis, and such treatment was shown in Ref. [43] to improve the crystallinity of the NCs. Therefore, we also applied similar post-synthesis heating to our solutions. Unexpectedly, we obtained severe deterioration of the NC quality, with the disappearance of the CZTS peak in the Raman spectra (Fig. 3a). In the spectra of CdTe/CZTS NCs, in addition, a strong PL band arose upon heating (two top curves in Fig. 3a). As the intensity of this band is higher in the samples with a larger concentration of CdTe seeds. No such band appears in the bare CZTS NCs (bottom spectrum in Fig. 3a); we may assume that this emission may be the PL of CdTe NCs, which is “recovered” due to destroying the CZTS overlayer by the heating. Note that the pristine CdTe NCs, which were used here as seeds for CZTS (over)growth, is strongly luminescent [15,44], but their PL is completely quenched upon CZTS phase overgrowth (Fig. 3a). There are two reasons for the complete quenching of the CdTe NC PL in the CdTe/CZTS NCs. The first reason can be a removal of the passivating ligand and introduction of non-radiative defect states at the formed interface with (intrinsically defective CZTS, as compared to CdTe). The second reason can be the transfer/capturing of the charge carriers photoexcited in the CdTe (core) to the CZTS (shell), where they recombine non-radiatively, as they do in bare CZTS NCs. Note, however, that the origin of the PL from the CZTS phase in the heated CdTe/CZTS NCs should not be discarded because a similar band is observed for the CZTS:Ag NCs sample as well (discussed in a separate section below). Although the AZTS compound is known to show PL [55], unlike CZTS one, additional experiments are needed to establish the origin of the PL in the CZTS grown on CdTe. One may assume, for instance, some charge or energy transfer mechanisms to be responsible for this emission via the excitation of CdTe NCs. But contrary to the as-synthesized CdTe/CZTS NCs, the CZTS phase can be less defective in heated CdTe/CZTS and thus does not quench the PL of the CdTe or re-emit it due to the charge of energy transfer from CdTe. Alternatively, the PL in all the samples may have a common origin but is not related with the semiconductor NC itself but with some products of the synthesis, which are common in all samples.

The CdTe and other seed NCs affect the structure of the CZTS phase as compared to pristine CZTS synthesis. Particularly, we systematically observed (not only for the representative set of samples presented in Fig. 3 but in other experiments performed to reproduce the effect) the main CZTS peak at lower frequency in CdTe/CZTS NCs, 326-329 cm^−1^, as compared to the pure CZTS NCs, 334-329 cm^−1^.

In numerous previous investigations of nano- [35,38,53,54] and microcrystalline [34,38,39,52] CZTS, the frequency below 332-334 cm^−1^ has been commonly interpreted as an indication of disordered cationic modification of the CZTS structure. It is, therefore, very interesting to observe here that this structure is preferably formed on the CdTe seed NCs.

**Fig. 4:**
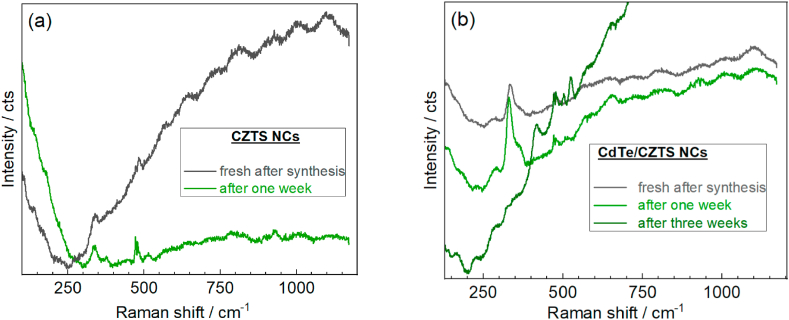
Raman spectra (λ_exc_ = 532 nm) of CZTS (a) and CdTe/CZTS (b) synthesized at 20°С.

An important factor affecting the final quality of the obtained CZTS NCs is the temperature at which the synthesis occurred. Thus, we observed that CZTS NCs synthesized at 10–14 °C (discussed above) exhibit a very good signal in the Raman spectra (CZTS spectrum in Fig. 3a), which indicates their good crystallinity, and the colloid remains stable for several months. If the synthesis is carried out at a higher temperature, for example, 20 °C, then there is both a deterioration in the quality of CZTS NCs, expressed in a decrease in the signal intensity in the Raman spectra, and an increase in the half-width of the band (Fig. 4a), and a decrease in the stability time of the colloids. For the synthesis temperature of 20 °C, the stability time decreases to about a month, and the CZTS NCs obtained at temperatures of ∼30 °C remain stable for no more than one to two weeks (Fig. 4a). However, even at such conditions, the samples with the addition of CdTe NPs remained stable longer, and their structural degradation began only in the third week (Fig. 4b). It is also worth noting that the same regularity is reflected not only in solutions but also in dried samples, the term of structural stability of which varied from more than six months to several weeks when the temperature at which the synthesis took place changed from 10 to 14 ^o^С to 30°С.

### 3.2 Discussion of the formation of the CZTS phase in presence of CdTe seed NCs

Chemical synthesis in aqueous solutions of metal salts is widely used to obtain a wide range of binary systems: PbSe, ZnSe, PbS, CdTe, etc., in the form of NC and polydisperse particles. However, the spread of these synthesis methods for forming NCs of quaternary compounds requires significant modification to ensure the possibility of obtaining structures with a stoichiometric composition, a narrow size distribution, and a predetermined morphology. The method of chemical synthesis in colloidal solutions consists in obtaining the initial solution of metal salts (in this case – SnCl_2_ or SnCl_4_, Cu(NO_3_)_2_, Zn(CH_3_COO)_2_) with the required ratio and their interaction with the chalcogenide in elemental form, in the form of a solution in an organic solvent or the form of a compound containing the necessary ion (in this case – Na_2_S). At the same time, the sequence of adding reagents may vary.

Generally, the particle formation for the ternary and quaternary NCs synthesis in an aqueous solution is similar to those described earlier for the binary compound CdTe. However, there are three cations in the system, so the reaction can occur in places of the highest concentration of Sn^2+^ or Sn^4+^, Cu^2+^, Zn^2+^ cations, that is, on the adsorption layers of these ions on the stabilizer molecule. However, the processes of adsorption of these cations on the stabilizer surface are competitive, so not only quaternary NCs with a stoichiometric composition but also secondary phases (binary and ternary compounds) can form.

Adding the CdTe nuclei to the colloidal solution may reduce the probability of the secondary phases formation. CdTe NCs have a clearly expressed negative charge and are characterized by a larger effective surface, as a result of which they are a much more thermodynamically favorable adsorption center for Sn^2+^ or Sn^4+^, Cu^2+^, Zn^2+^ cations than the stabilizer molecule. Therefore, in the system, the processes of chaotic adsorption of cations on individual molecules of the stabilizer with their subsequent interaction with each other and anion ions do not occur in the system. However, the adsorption of ions on the surface of CdTe NCs with subsequent chemical interaction in the adsorption layer is observed. Therefore, we cannot also rule out the possibility that colloidal CZTS NCs at the time of their formation on the surface of a foreign seed have an amorphous structure. However, the transition from the amorphous to the crystalline phase occurs due to the greater stability of the crystalline state during the aging process or heat treatment. The time of transition of an amorphous structure into a crystalline one largely depends on the nature and amount of the stabilizer. Further growth of crystal nuclei takes place by direct embedding of ions into the surface of the crystal under the action of the field of long-range forces of the crystal lattice, which exert an orienting influence on them. In this way, the formation of crystalline CdTe/CZTS NCs takes place.

### 3.3 CZTS NCs with cation substitution for Ag or Ba

According to the valency, Ba is supposed to substitute Zn in the CZTS lattice. However, because of the multiple valencies of copper, Cu^+^/Cu^2+^, and related to the phenomenon of Cu–Zn cation disorder (antisite defects) [33,42], the mechanism of occupation of the lattice sites with Ba may also be complicated. And this assumption is indeed confirmed by the Raman results obtained in this work.

**Fig. 5:**
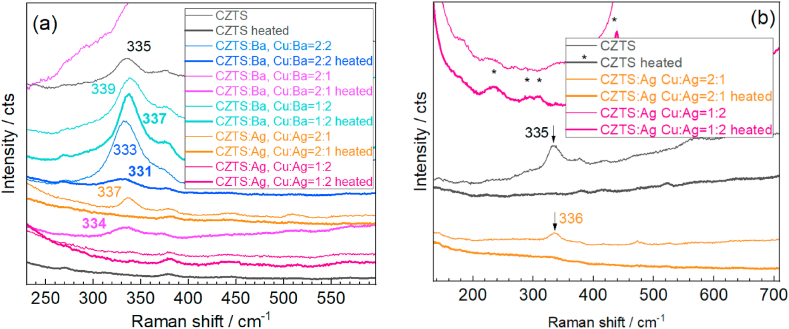
Raman spectra of CZTS, CZTS:Ag, and CZTS:Ba in solution (a) and on a dried film (b). Thin curves correspond to as-synthesized samples, and thick curves of the same color – corresponding sample subject to post-synthesis heating.

Three NC samples were prepared in this work to investigate the effect of cationic substitution with Ba, with the nominal ratio of Cu:Ba:Zn equal to 2:2:1, 1.5:0.7:1, and 0.7:1.5:1. Pure CZTS NCs were investigated for comparison. Raman spectra of the whole set of the NCs are presented in Fig. 5. The samples with Cu:Ba ratios 2:2 and 1.5:0.7 exhibited Raman bands stronger than that of pure CZTS NCs. Upon heating, the peak decreased in the sample with the highest Ba content, while it got even stronger and sharper for the intermediate one. For the lowest Ba content, the as-synthesized NCs did not reveal a Raman peak, only a strong PL signal (the top curve in Fig. 5a), but the vibrational fingerprint of the crystalline structure was detected upon heating, most likely due to dropped PL background. Although at first glance, the behavior of the NCs with the lowest Ba content may appear opposite to that of the other two samples, with higher Ba content, there is a clear general trend for all three samples. Namely, more CZTS forms in the as-synthesized NCs at higher Ba content, while its stability towards post-synthesis heat treatment is better for lower Ba content. The latter structure also exhibits a higher degree of cation order, as revealed by the higher peak frequency compared to CZTS:Ba with higher Ba content and pure CZTS NCs. It is reasonable to assume a direct relationship between the higher degree of cation order and better thermal stability of the NCs.

Interesting is also the behaviour of the phonon peak position of CZTS:Ba NCs samples. Compared to the pure CZTS NCs, its frequency is higher for the highest and lowest Ba content and slightly lower than CZTS frequency for the intermediate Ba content. From this preliminary Raman study of a limited set of NC samples, one may conclude that the sample with intermediate Ba content is, in a certain sense, an optimal sample in terms of structural quality in an as-synthesized state and its further improvement upon heating. Making such a conclusion we assume that in analogy to pure CZTS, the high phonon frequency indicates better crystalline quality. It should be noted that in the literature reports, the main Raman peak position in Cu_2_BaSnS_4_ varies from 341 cm^−1^ [10] up to 345 cm^−1^ [56]. In Ref. [19], two equally strong Raman bands were reported, at 300 cm^−1^ and 343 cm^−1^, respectively, but the origin of the additional band was not commented on. Nevertheless, there is a general agreement among the literature reports that the main peak position in Cu_2_BaSnS_4_ occurs at a higher frequency than in Cu_2_ZnSnS_4_. Therefore, our NC sample with the intermediate Ba content may have a more ordered (cationic) lattice than the other two samples, with higher and lower Ba content. It should be noted, however, that in the discussed three samples not only Ba content was varied but also Cu content. But also from this point of view, we can draw a conclusion that is in qualitative agreement with the literature reports. In particular, the sample with intermediate Ba content, for which the best structural quality among the three samples is inferred based on the Raman data, is the sample with the lowest Cu content. And it is known from numerous previous studies of CZTS compounds that low Cu content is beneficial for the good structural quality of the CZTS lattice, while Cu excess can lead to deuteriation of the CZTS lattice, as well as to segregation of the Cu_x_S impurity phase [35,38].

Since the results obtained, one can conclude a rather complex effect of Ba incorporation into the CZTS lattice on its structural stability, and this effect needs a separate detailed investigation. Nevertheless, we can also conclude that this type of cation substitution can be an efficient tool for tuning the structural properties of CZTS-like NCs and hetero-NCs.

As to the NCs formed with adding Ag ions, we can see that for Cu:Ag ratio of 2:1 a weak but sharp Raman peak is observed in the as-synthesized sample at a higher frequency than the pure CZTS peak, which is an expected direction of the shift due to incorporation of Ag into the CZTS lattice [[20], [21], [22],^55^,^57^]. On the contrary, no peak in the NCs subject to heating is observed. Only several higher-frequency features appear, which are neither present in the spectra of other samples nor correspond to any of the known secondary phases of CZTS or AZTS [22], so they might be related to some by-products of the synthesis. For the sample with high nominal Ag content, Cu:Ag = 1:2, no Raman peak is observed in both spectra. This result aligns with our previous works on aqueous Ag_x_Cu_1-x_ZnSnS_4_ NCs synthesized at similar mild conditions [21,22], where severe deterioration of the CZTS lattice was observed at high Ag contents.

All the trends and effects observed in the Raman spectra of the solutions are qualitatively reproduced in the measurements performed on corresponding dried films (Fig. 5b).

## 4 Conclusions

By using Raman spectroscopy as a main structural characterization tool, we have investigated the series of NCs based on Cu_2_ZnSnS_4_-like compound from as-synthesized solutions and films obtained by drop-casting method onto a glass substrate. Raman data are corroborated by optical absorption measurements and transmission electron microscopy on selected samples. In particular, we have established that the method of affordable colloidal synthesis developed in this work allows obtaining not only bare CZTS NCs but also cation-substituted NCs, and CZTS-based hetero-NCs. For the latter, the synthesized in advance NCs of another material are introduced into the reaction solution so that the formation of CZTS phase takes place preferably on these “seed” NCs. The CdTe and Ag NCs are found to be good seed NCs, resulting in a comparable or even better quality of the CZTS phase than bare CZTS NCs. For Au NCs, on the contrary, no hetero-NCs could be obtained under the given condition. Partial substitution of Zn for Ba during the synthesis of CZTS NCs results in a superior structural quality of (CZTS:Ba) NCs in a specific composition. In contrast, the introduction Ag for partial substitution of Cu deteriorates the structural quality of the (CZTS:Ag) NCs obtained.

## Author contribution statement

Olga A. Kapush: Conceived and designed the experiments.

Volodymyr Dzhagan, Ivan S. Babichuk, Volodymyr O. Yukhymchuk: Wrote the paper.

Nazar V. Mazur, Mykola I. Danylenko, Yevhenii O. Havryliuk: Performed the experiments.

Anatoliy Karnauhov, Roman A. Redko, Serhiy I. Budzulyak: Analyzed and interpreted the data.

Serhiy Boruk: Contributed reagents, materials, analysis tools or data.

## Data availability statement

Data will be made available on request.

## Declaration of competing interest

The authors declare that they have no known competing financial interests or personal relationships that could have appeared to influence the work reported in this paper.
